# Antimicrobial Nitric Oxide-Releasing Electrospun Dressings for Wound Healing Applications

**DOI:** 10.1021/acsmaterialsau.1c00056

**Published:** 2022-01-25

**Authors:** Man Li, Jenny Aveyard, Kyle G. Doherty, Robert C. Deller, Rachel L. Williams, Keli N. Kolegraff, Stephen B. Kaye, Raechelle A. D’Sa

**Affiliations:** † School of Engineering, University of Liverpool, Liverpool L69 3GH, United Kingdom; ‡ Department of Eye and Vision Science, Institute of Life Course and Medical Science, University of Liverpool, Liverpool L7 8TX, United Kingdom; § Department of Plastic and Reconstructive Surgery, The Johns Hopkins University School of Medicine, 601 North Caroline Street, Baltimore, Maryland 21287, United States

**Keywords:** wound healing, nitric oxide, polycaprolactone, gelatin, antimicrobial activity, electrospinning

## Abstract

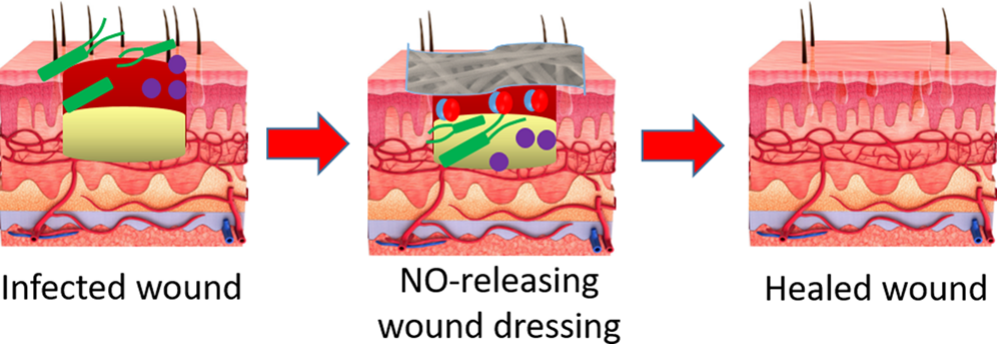

Nonhealing and chronic wounds represent a major problem for the quality of life of patients and have cost implications for healthcare systems. The pathophysiological mechanisms that prevent wound healing are usually multifactorial and relate to patient overall health and nutrition, vascularity of the wound bed, and coexisting infection/colonization. Bacterial infections are one of the predominant issues that can stall a wound, causing it to become chronic. Successful wound healing often depends on weeks or months of antimicrobial therapy, but this is problematic given the rise in multidrug-resistant bacteria. As such, alternatives to antibiotics are desperately needed to aid the healing of chronic, and even acutely infected wounds. Nitric oxide (NO) kills bacteria through a variety of mechanisms, and thus, bacteria have shown no tendency to develop resistance to NO as a therapeutic agent and therefore can be a good alternative to antibiotic therapy. In this paper, we report on the development of NO-releasing electrospun membranes fabricated from polycaprolactone (PCL)/gelatin blends and optimized to reduce bacterial infection. The NO payload in the membranes was directly related to the number of amines (and hence the amount of gelatin) in the blend. Higher NO payloads corresponded with a higher degree of antimicrobial efficacy. No cytotoxicity was observed for electrospun membranes, and an *in vitro* wound closure assay demonstrated closure within 16 h. The results presented here clearly indicate that these NO-releasing electrospun membranes hold significant promise as wound dressings due to their antimicrobial activity and biocompatibility.

## 1 Introduction

Chronic nonhealing wounds represent a substantial global problem for the quality of life of patients and treatment options for clinicians and healthcare workers. In the UK alone, it is estimated that 3.8 million patients are undergoing treatment by the National Health Service (NHS) and the number of chronic wounds is rising at a rate of 12% per year.^1^ Wound management costs the NHS £8.3 billion, which can be broken down into £2.7 billion for healed wounds and £5.6 billion for unhealed wounds. A wound can become chronic (unhealed) when it gets stalled in one of the wound healing stages, such as prolonged inflammation.^1^ This prolonged inflammation stage may be due to infection, or the wound could also become highly susceptible to infection when in this state. Depending on the severity of the wound, local wound care can involve cleaning with saline and application of special dressings, bandages, or gauze. While these dressings can provide the necessary barrier protection from external factors such as dirt and microorganisms, they are unable to fight existing infections or actively facilitate in wound healing. As such, there is an unmet clinical need for a wound dressing that can treat and reduce the risk of infection and promote cellular regeneration, thereby kick-starting the wound healing process for chronic wounds.

Currently, the gold standard in antimicrobial wound care, without using antibiotics, involves the use of silver-based dressings.^2^ While these dressings have made great strides in reducing microbial bioburden, they do not actively promote wound healing.^3^ NO is a promising alternative to antibiotics as it has shown potent antibacterial activity. As it has many mechanisms of action against bacteria, it is difficult for bacteria to develop resistance against this multipronged assault.^4,5^ NO is endogenously produced and has several physiological functions that are concentration dependant. Endogenously produced NO ranges from 1 nM to 1 μM^6^ and is involved in infection control, promotion of angiogenesis, collagen deposition, and re-epithelialization.^7−10^ Therefore, exogenously produced NO-releasing materials can be formulated to have a dual role in infection control and wound repair.

NO is a highly reactive lipophilic and hydrophilic free radical with a short half-life and an activity radius of 100 μm from its origin.^11^ Consequently, NO must be generated rapidly at the site of action in the dose required to have its intended therapeutic effect. As NO is a gas, practical delivery of the gas to a wound is problematic, although it has been investigated within this remit.^12,13^ Delivery of NO by tethering of NO donors to polymeric dressings represents a promising solution to treat infected wounds. There are numerous NO-releasing coatings and materials currently under investigation, many of which have demonstrated efficacy against bacterial infections.^14−16^

Electrospinning has developed significant traction for the manufacture of advanced dressings. These nanofibrous membranes can be fabricated with a variety of polymers to have key features such as high surface area, porous structures, and gas permeation that make them ideal for wound-healing applications.^17−20^ The incorporation of antimicrobial agents or therapeutics into these membranes and the ability to spin the membranes into any shape and size means that these materials can potentially be used for personalized bandages for virtually any wound. The structure of electrospun membranes can also promote the absorption of wound exudate, which can decrease the risk of wound infections.^21^ Balkus and co-workers have previously fabricated NO-releasing electrospun polyacrylonitrile bandages that enhance wound-healing progression when applied to wounds in weekly and daily applications.^22^ Zhao et al. have successfully demonstrated that electrospun NO-releasing PCL/chitosan wound dressings can speed up the wound-healing process by enhancing re-epithelialization and granulation formation.^23^

**Figure 1:**
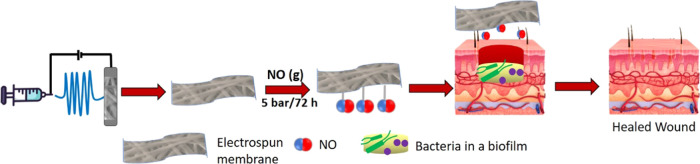
Schematic representation of fabrication of NO-releasing electrospun PCL/gelatin membranes.

In this paper, we report on the fabrication of five blend ratios of electrospun PCL/gelatin membranes that have tethered with NO for its roles in reducing infection and wound repair, as shown schematically in Figure 1. PCL is a biocompatible and bioresorbable polymer that has regulatory approval for a number of medical devices and has a long history in the development of wound dressings. It is currently classified as a compatible bioresorbable material for soft and hard tissue.^23^ Although electrospun PCL membranes have excellent mechanical properties that make them widely exploited for wound dressing applications, the inherently high hydrophobicity prohibits proactive biological responses.^24^ Gelatin is a naturally occurring biopolymer derived from collagen. It is biocompatible and nonimmunogenic but has low mechanical strength and a fast degradation rate.^25^ By combining PCL and gelatin into a composite material, it can benefit from the advantages of both polymers: mechanical strength, degradation, and biocompatibility. In this study, blended electrospun PCL/gelatin nanofibers were fabricated, followed by chemical crosslinking with genipin to further increase the mechanical properties and structural integrity. Varying ratios of PCL/gelatin were investigated to obtain the optimal formulation in terms of NO loading, antimicrobial efficacy, and cytocompatibility. The antimicrobial efficacy was evaluated against Gram-positive (*Staphylococcus aureus*) and Gram-negative (*Escherichia coli*) bacteria in a planktonic and biofilm-adhered state. The biocompatibility and the wound-healing efficiency were assessed via a resazurin assay and an *in vitro* wound closure assay.

## 2 Materials and Methods

### 2.1 Materials

Gelatin (gel strength 300 g Bloom, Type A), poly(ε-caprolactone) (PCL) (*M*_n_: 80 000 g·mol^–1^), genipin (≥98%), 2,2,2-trifluoroethanol (TFE), Luria-Bertani (LB) broth, agar, DMEM/F12, penicillin and streptomycin, biopore membrane filter roll, and hydrophilic PTFE were acquired from Merck (Gillingham, U.K.) and used as received. The human keratinocyte HaCaT cell line^26^ was kindly donated by Dr. Kevin Hamill (University of Liverpool, U.K.), and the WS1^27^ human skin fibroblast cell line (ATCC CRL-1502) was purchased from American Type Culture Collection (Manassas, VA). Fetal bovine serum was acquired from Biosera (Nuaille, France). Zinc diethyldithiocarbamate (ZDEC) was purchased from Japanese Food and Drug Safety Centre (Hadano, Japan). All common, analytical grade laboratory solvents and salts, including ethanol, acetic acid (HAc), hydrochloric acid (HCl), disodium phosphate, sodium acetate (NaAc), and sodium salt were from Sigma-Aldrich and used as received.

### 2.2 Electrospinning

The polymer solutions of PCL and gelatin were prepared separately in TFE (10 wt %) by stirring at room temperature overnight. After both polymers were dissolved completely in the solvent, the two solutions were blended at weight ratios (PCL solution/gelatin solution) of 75:25, 50:50, and 25:75. The mixture was turbid because of the PCL and gelatin has a tendency to phase separate. Consequently, a few drops of HAc (<0.3%) were added to the solution followed by further stirring (4 h) to obtain a homogeneous PCL/gelatin solution for electrospinning.^28^

Electrospinning was carried out using a climate-controlled electrospinner (IME, Waalre, Netherlands). The five prepared solutions were stirred for 4 h and then transferred into a 5 mL plastic syringe equipped with a needle with a blunt end (diameter = 0.4 mm, reciprocating motion speed of 10 mm/s over a distance of 50 mm) and the polymer solution flow rate was set to of 2 mL/h. A cylindrical grounded mandrel (diameter = 90 mm, height = 180 mm) covered with aluminum foil was set at a distance of 185 mm away from the needle tip and rotated at 200 rpm. A high voltage of 18–20 kV was applied between the needle and the collector. Membranes were collected after electrospinning for 120 ± 10 min in a climate control system with a relative humidity of 70% and temperature of 23 °C. The electrospun membranes were vacuumed dried for residual solvent removal.

### 2.3 Crosslinking of Electrospun Membranes

**Scheme 1:**
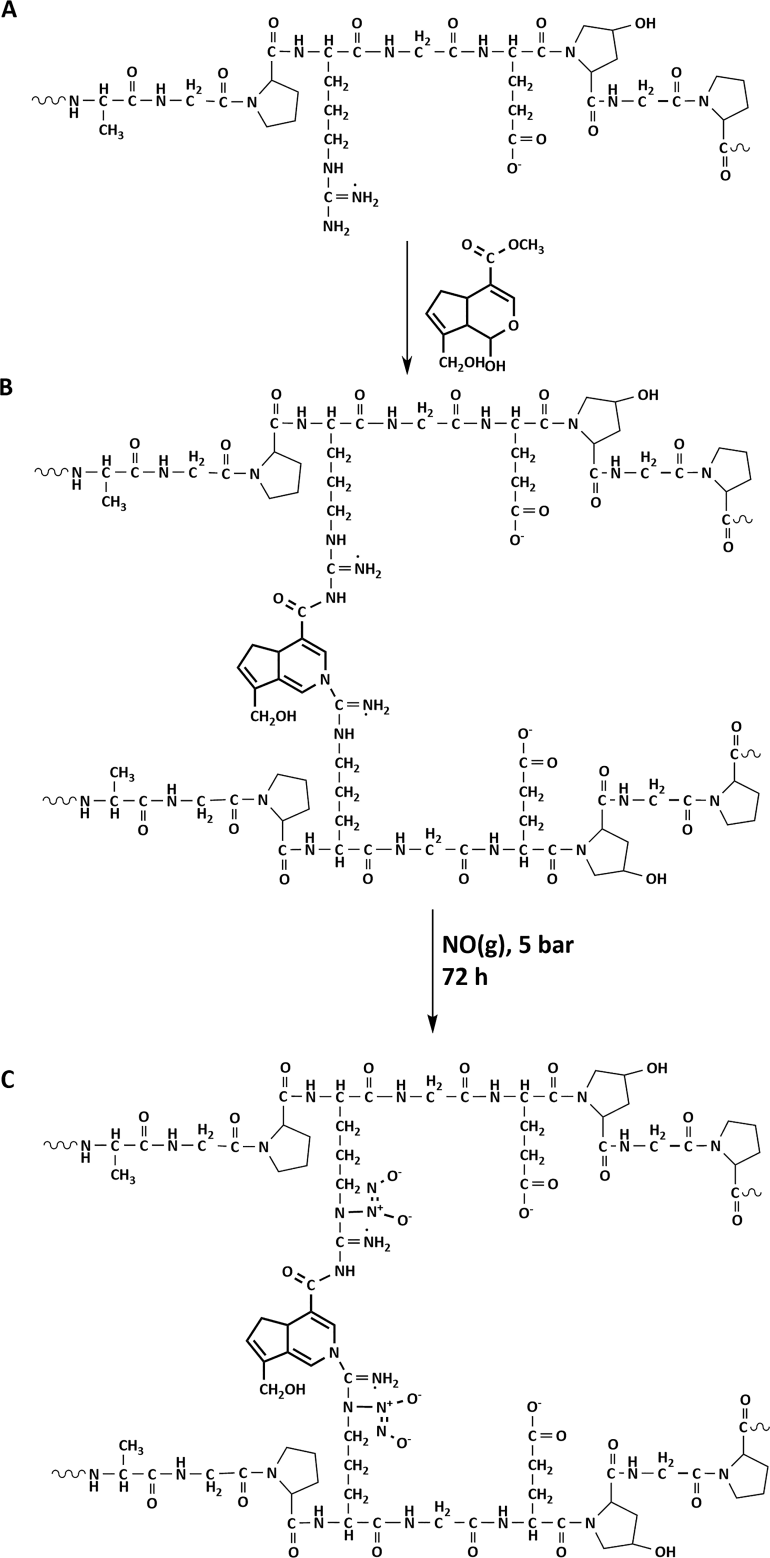
(A) Gelatin Chemical Structure; (B) Genipin Crosslinking Reaction on Primary Amines; and (C) Formation of *N*-Diazeniumdiolates on Crosslinked Gelatin under High NO Pressure of 5 bar for 72 h

The electrospun membranes (P, G, P25, P50, P75) were crosslinked for 6 days by soaking in 0.2% (w/v) genipin/ethanol solution. Following crosslinking, these scaffolds (P, G, P25, P50, and P75) were washed with adequate ethanol 3 × 10 min to remove residual genipin. The crosslinked membranes were dried in vacuum for 3 days to eliminate any leftover solvent. All of the scaffolds were stored in a vacuum desiccator covered with foil until use to prevent degradation. The crosslinking mechanism of genipin (Scheme 1A,B) includes two modes of action with different sites on the genipin.^29^ The first mechanism of action involves nucleophilic substitution reaction between the −NH_2_ and −COOCH_3_, creating a secondary amide bond and replacing the ester group. The second action involves nucleophilic attack of the primary amine on gelatin with the C3 carbon on genipin, generating an intermediate aldehyde functional group. A heterocyclic compound is then formed by the interaction of the newly formed secondary amine with the aldehyde group.

The electrospun PCL/gelatin membranes had thickness measurement carried out using a microcaliper (Mitutoyo, Japan). Apparent density and porosity measurements were calculated as follows^30^
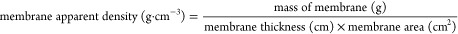
1
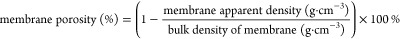
2

### 2.4 Tethering of NO Donors

Electrospun membranes were functionalized with the NO donor, *N-*diazeniumdiolate (Scheme 1C), in a NO reactor that has been built in-house as previously reported.^15^ Briefly, the chamber of the reactor was purged with argon at 6 bar pressure (BOC, Guildford, U.K.) for 5 min (3×) and 10 min (3×). This was carried out to ensure that all traces of oxygen and water were removed from the reaction. NO gas (BOC, Guildford, U.K.) was then charged into the chamber at a pressure of 5 bar over a 72 h period. After this 72 h period, unreacted NO gas was flushed out of the reactor by purging the system with argon at 6 bar pressure for 5 min (2×) and 10 min (2×). This process resulted in the formation of *N*-diazeniumdiolate-functionalized membranes, which were stored in the freezer at −20 °C prior to any analysis. As PCL has no amine group in the backbone of the polymer, *N*-diazeniumdiolates cannot be formed on this polymer, and as such, it is the control group in NO release tests. The nomenclature for samples tethered with NO has a suffix of /NO: P/NO, P25/NO, P50/NO, P75/NO, and G/NO. A table outlining the nomenclature is given in Table 1.

**Table 1:** Nomenclature for Various Electrospun Membranes

name	PCL wt %	gelatin wt %	sample name with NO
P	100	0	P/NO
P75	75	25	P75/NO
P50	50	50	P50/NO
P25	25	75	P25/NO
G	0	100	G/NO

### 2.5 Morphological of Electrospun PCL/Gelatin Membranes

The morphology of the membranes was examined by scanning electron microscopy (SEM). Samples of the membranes were cut to 10 × 10 mm^2^ sizes and coated with gold (Q150T ES sputter coater; Quorum, East Sussex, U.K.). Images were captured on a JSM 7001F field emission scanning electron microscope (JEOL, Tokyo, Japan). The diameter analysis of fibers was performed by randomly picking up at least 115 fibers from each material enlargement SEM images and measuring in ImageJ.

### 2.6 Characterization of Membranes

The wettability of the surface of the membranes was investigated via a sessile drop method using an Attension ThetaLite equipped with OneAttension software (Biolin Scientific, Västra Frölunda, Sweden). The image began to be captured from when the drop contacted with the surfaces and was continuously captured for 10 s. All contact angle measurements were made in triplicate.

The Fourier-transform infrared spectroscopy (FTIR) spectra of electrospun membranes were analyzed using a PerkinElmer frontier IR system over the range of 500–4000 cm^–1^ at a scanning resolution of 4 cm^–1^.

Mechanical testing was conducted as previously published.^31^ Membranes were cut into rectangular samples with a dimension of 3 cm × 1 cm. The membranes were secured with sticky tape over a paper window, and this gave the final dimension of the analyzed section of 2 cm × 1 cm. Paper windows were used as this enabled ease of positioning and handling of the sample within the tensile grips. Before beginning, the sides of the windows were cut to make sure only the membrane was loaded for testing. An electronic micrometer was used to study the thickness of the sample membranes. A UniVert (CellScale, Waterloo, Ontario, Canada) was used to carry out the measurements. Measurements were obtained with the instrument in tensile mode with a 10 N load cell and a strain rate of 400%/min. The 100% PCL sample could not be tested as the actuators could not separate enough. The 100% gelatin samples were too brittle to load into the grips.

The *in vitro* degradation properties of the NO-releasing membranes were evaluated by individually immersing 1 cm × 1 cm of each samples (initial mass weighed prior) into 1 mL sterilized PBS at 37 °C in Eppendorf tubes. At time points of 1, 2, 4, and 25 days, samples were taken out, washed with DI water, and oven-dried at 55 °C for overnight before weighing. The percentage of mass remaining was calculated as follows
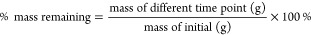
3

### 2.7 NO Loading

The payload and rate of release from the NO-releasing membranes were investigated using a Sievers 280i Chemiluminescence Nitric Oxide Analyzer (NOA280i, GE). The membranes (10 × 10 mm^2^) were placed in a three-neck round-bottom flack and soaked in 5 mL of acetic acid buffer (pH 4), phosphate-buffered saline (PBS; pH 7.4), or cell culture media at ambient temperatures. The flask was constantly purged with nitrogen gas at a rate of 200 mL/min on the NOA, which matches the collection rate. The NOA was connected to a vacuum pump that extracts the gases into the reaction cell and keeps the pressure constant. The rate and concentration of NO release were measured at 1 s intervals for over 20 h. Measurements were all carried out in triplicate.

### 2.8 Antimicrobial Assays

#### 2.8.1 Biofilms Colony Forming Unit (CFU) Assay on Adhered Bacteria

The antimicrobial efficacy of the NO-releasing membranes was investigated against *E. coli* ATCC 10536 and *S. aureus* ATCC 25923. Overnight cultures of *E. coli* and *S. aureus* were diluted to 1 × 10^6^ CFU/mL in LB broth, according to the absorbance @600 nm and a 0.5 McFarland Standard.^32^ Membranes (10 × 10 mm^2^) were placed in a 24-well plate. Each of the sample wells was charged with diluted bacterial solution (1 mL) and incubated at 37 °C for bacterial colonization and biofilm formation to occur. Membranes were washed with PBS gently at 4 and 24 h time points of incubation. This was to remove any planktonic bacteria which were not attached. Finally, a fresh aliquot of 1 mL of LB broth was used to detach and resuspend biofilms. Bacterial CFU counts were calculated using the Miles and Misra method following serial dilutions on LB agar plates.^33^ The experiment was repeated at least three times with three technical replicates.

#### 2.8.2 Antimicrobial Activity against Planktonic Bacteria

The overnight cultures of *E. coli* and *S. aureus* were diluted to 1 × 10^6^ CFU/mL in LB broth. Membranes (10 × 10 mm^2^) were stored in a 24-well plate. Each of the sample wells was charged with diluted bacterial solution (1 mL) and incubated at 37 °C for bacterial growth. After 4 and 24 h incubation, the liquids in the well plates were removed to another aseptic well plate and serially diluted with LB broth. The antimicrobial activity against planktonic bacteria was determined on LB agar plates using the Miles and Misra method.^33^ The experiment was repeated at least three times with three technical replicates.

### 2.9 Cell Viability and Wound Gap Closure Efficacy

#### 2.9.1 Cytotoxicity

Human keratinocyte HaCaT^26^ and WS1^27^ human skin fibroblast cell lines (ATCC CRL-1502) were cultured at 37 °C and 5% CO_2_ in DMEM/F12 (Merck, Gillingham, U.K., D8437) and supplemented with 10% fetal bovine serum, 100 U/mL penicillin, and 10 μg/mL streptomycin.

Extracts of electrospun materials and controls were made by incubating materials in the supplemented cell culture media (DMEM/F12, 10% FBS, 100 U/mL penicillin, and 10 μg/mL streptomycin) for 24 h at 37 °C as per ISO 10993-12. Biopore membrane filter roll, Hydrophilic PTFE, 40 μm thickness, was used as a negative, noncytotoxic control and polyurethane film containing 0.1% zinc diethyldithiocarbamate (ZDEC) served as a positive, cytotoxic control. HaCaT and WS1 cells were seeded in 96-well plates (Corning Costar, Flintshire, U.K.) at 1 × 10^4^ cells/well and incubated for 24 h, after which growth media were removed from wells and 100 μL extract media from materials, controls, and plain vehicle control media were added to the cells. Cells were incubated for a further 24 h. A 10× stock resazurin sodium salt solution of 1 mg/mL in PBS was prepared and sterile filtered. After the 24 h incubation, the wells were emptied and blotted dry; then, 150 μL of the resazurin working solution, diluted in complete media, was added to wells including cell-free blank wells. After 4 h of incubation at 37 °C, 100 μL of resazurin solution was transferred into 96-well black plates. The fluorescence was read using 544 nm excitation and 590 nm emission filters on FluorSTAR Optima Plate Reader (BMG Labtech, Aylesbury, U.K.). Percentage viability was calculated using the equation below, where sample reading is the fluorescence of samples, positive and negative controls, and VC reading is the fluorescence of the vehicle control. Materials are considered noncytotoxic if the % viability is greater than 70% in accordance with ISO10093-5.
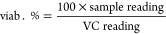
4The experiment was repeated three times with six technical replicates.

#### 2.9.2 *In Vitro* Wound Gap Closure

HaCaT cells were transfected via a lentiviral vector incorporating green fluorescent protein (GFP) and puromycin resistance. Successfully transfected cells were selected for by adding 5 μg/mL puromycin to complete medium for 24 h and imaged using a ZEISS Apotome.2 fluorescence microscope (Carl Zeiss Ltd., Cambridge, U.K.) under live cell conditions to assess fluorescence. Transfected cells were then serially diluted to isolate a single monoclonal population of cells for further use.

HaCaT-GFP cells were seeded at 7 × 10^4^ cells/well into ibidi 2-well culture inserts (Ibidi, Martinsried, Germany). After 6 h of incubation at 37 °C, inserts were carefully removed and cells were rinsed. The gap was imaged using a ZEISS Apotome.2 fluorescence microscope with a 10× objective at 30 min time points over a 16 h time period (Zen 2 v2.0, Carl Zeiss Ltd., Cambridge, U.K.). Due to the limitation of well numbers for image capturing, only P25, P50, P75, P25/NO, P50/NO, and P75/NO have been tested. The gap closure was measured using the Wound_Healing_Size plugin^34^ for Image J (NIH, Bethesda, Massachusetts). The experiment was repeated three times with three technical replicates.

### 2.10 Statistical Analysis

One-way analysis of variance (ANOVA) with the Tukey method was used to analyze the differences among the variously treated samples. All data were collected in triplicate and displayed as mean ± standard deviation. A value of *p* < 0.05 was taken as being statistically significant.

## 3 Results and Discussion

The feasibility of developing NO-releasing electrospun membranes for antimicrobial and wound-healing capabilities was investigated. Five formulations of membranes consisting of varying ratios of PCL/G were electrospun, followed by crosslinking with genipin. A schematic illustration of the process is given in Figure 1.

### 3.1 Morphology of PCL/Gelatin Electrospun Membranes

**Figure 2:**
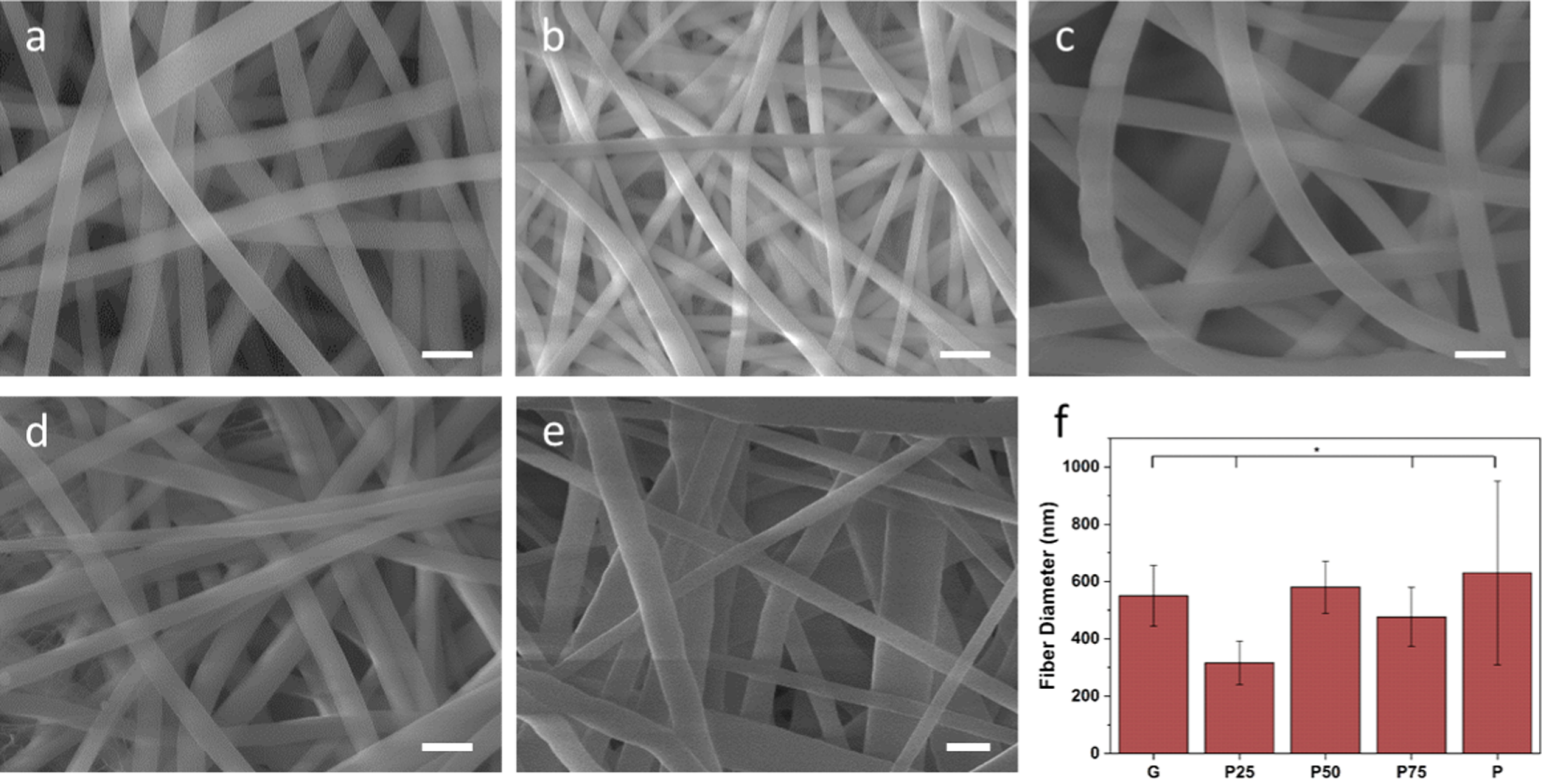
Scanning electron microscopy (SEM) of crosslinked electrospun PCL/G membranes: (a) G, (b) P25, (c) P50, (d) P75, (e) P, and (f) average diameters. The scale bar represents 1 μm in figures. *p < 0.05.

The SEM micrographs of genipin-crosslinked nanofibrous membranes are shown in Figure 2a–e. The electrospun membranes had a randomly interconnected structure with a smooth ribbon-like morphology. The fiber diameters for the five formulations varied from 316 to 630 nm are shown in Table 2; the diameter distribution is given in Figure S1. The gelatin sample has an average fiber diameter of 550 ± 106 nm (Figure 2a). Upon addition of increasing ratios of PCL to polymer blend solutions, the fiber diameters varied but remained smooth in morphology (Figure 2b–d). The samples P25, P50, and P75 showed average fiber diameters of 316 ± 76, 580 ± 91, and 476 ± 103 nm, respectively. The pure PCL electrospun membranes had a fiber diameter of 630 ± 320 nm, which is significantly higher than other fibers. The fiber diameter increase of pure PCL can be attributed to the higher viscosity of the precursor solution compared to when gelatin is in the blend.^35,36^ The charge density of the surface of the solution jet is increased due to the ionization of the gelatin in the precursor solution. This increases the conductivity and self-repulsion properties of the jet, which in turn creates fibers with narrower diameters.^37^

**Table 2:** Fiber Contents and Fiber Diameter of Crosslinked Electrospun Membranes

sample	gelatin wt %	PCL wt %	fiber diameter (nm)	apparent density (g/cm^3^)	porosity %
G	100	0	550 ± 106	0.55 ± 0.04	51 ± 3
P25	75	25	316 ± 76	0.18 ± 0.01	83 ± 1
P50	50	50	580 ± 91	0.24 ± 0.06	78 ± 5
P75	25	75	476 ± 103	0.27 ± 0.02	76 ± 2
P	0	100	630 ± 320	0.23 ± 0.01	79 ± 1

### 3.2 Apparent Density and Porosity Evaluation

The apparent density and porosity are important parameters for wound dressing applications as ideally the material should allow for sufficient gas and nutrient exchange, support cell proliferation on the wound but prevent cell ingress into the dressing.^30^ The apparent density of various PCL/G electrospun membranes, G, P25, P50, P75, and P are given in Table 2. Pure gelatin, G, had an apparent density of 0.55 g/cm^3^. The introduction of PCL to the blend lowered the apparent density to 0.18, 0.24, 0.27, and 0.23 g/cm^3^ for the P25, P50, P75, and P samples, respectively. The bulk density of the PCL/G membranes (1.2–1.3 g·cm^–3^) combined with apparent density calculations was used to determine its porosity with the results presented in Table 2. The porosity of all of the electrospun membranes with PCL had a significantly higher porosity in comparison with G. Pure gelatin, G had a calculated porosity of 51%, and P25, P50, P75, and P samples had porosities of 83, 78, 76, and 79%, respectively. All of the electrospun membranes with PCL components exhibited porosity at a high range.

### 3.3 Wettability: Contact Angle

**Figure 3:**
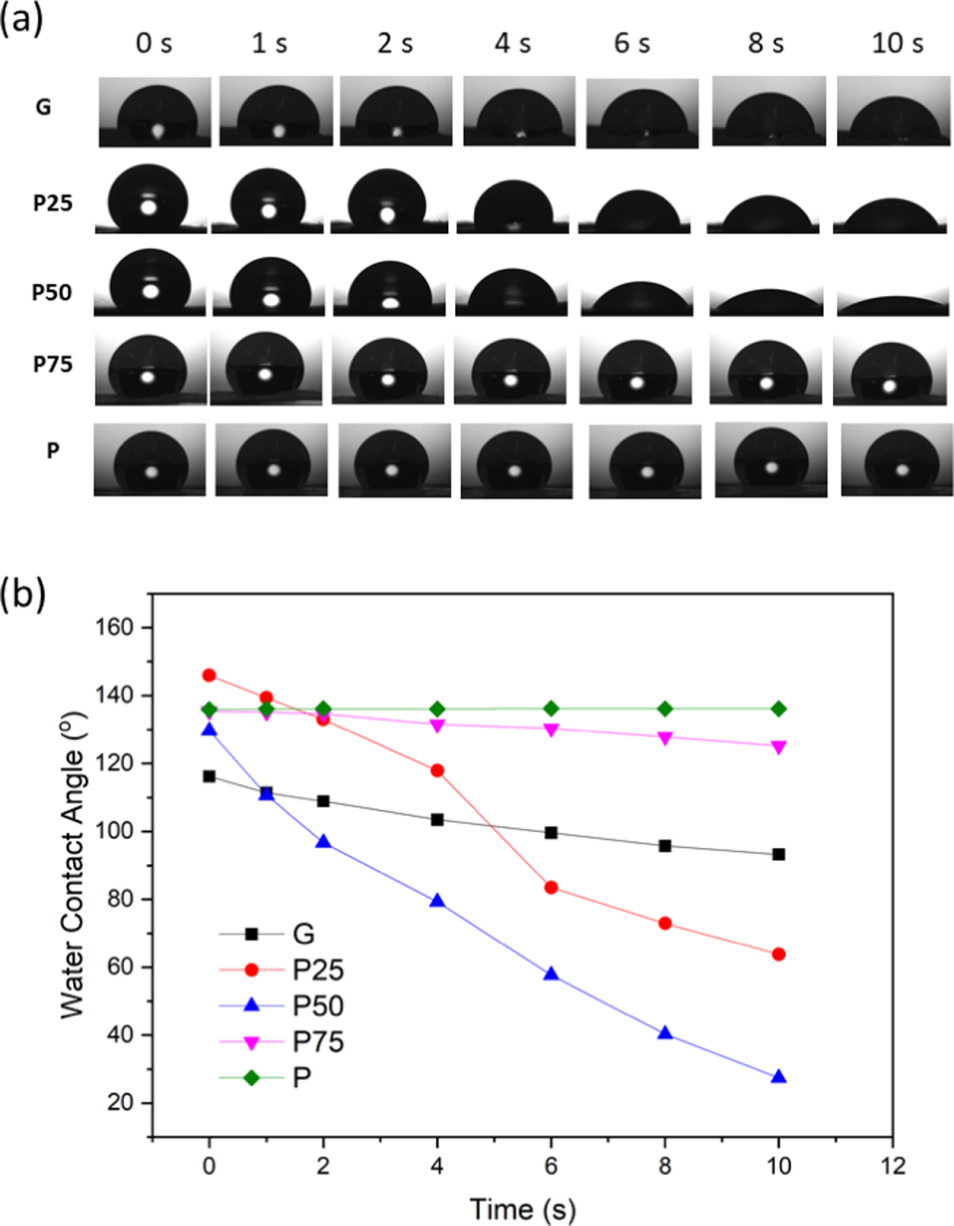
(a) Optical water contact angle images of G, P25, P50, P75, and P electrospun membranes and (b) measured dynamic contact angle of water droplets on the membrane surfaces for the initial 10 s. Representative image of each sample selected from at least triplicates taken.

Water absorption of electrospun membranes is dictated by the hydrophilicity and porosity of the membranes. This is an important parameter for a wound that are highly exudative as excess exudate can delay wound healing.^38^ Surface hydrophilicity and water absorbability of electrospun membranes have been characterized by water contact angle (WCA) measurement (Figure 3). The pure gelatin membrane, G, had an initial WCA of 116°, which decreased to 93° after contacting with a water droplet for 10 s. This could be explained by the low porosity of G inhibiting the water penetration at the first 10 s contacting given its lower porosity compared with all of the other membranes. The pure PCL membrane, P, was hydrophobically displaying a WCA of ∼136° during the whole measurement process. The mixture membranes P25, P50, and P75 displayed different WCA. P25 and P50 presented high initial WCA of 146 and 130°, respectively, and both rapidly reduced to 64 and 27° at the 10 s. The increased hydrophilicity of P25 and P50 is due to the high porosity and the hydrophilic carboxyl functional groups in the gelatin chemical structure.^35^ This implies that a higher mass percentage of gelatin in hybrid fiber membranes improves the hydrophilicity. As the hydrophobicity of the membrane was dominated by PCL, the WCA of P75 slowly decreased from 135 to 125° in 10 s.

### 3.4 Chemical Evaluation: FTIR

**Figure 4:**
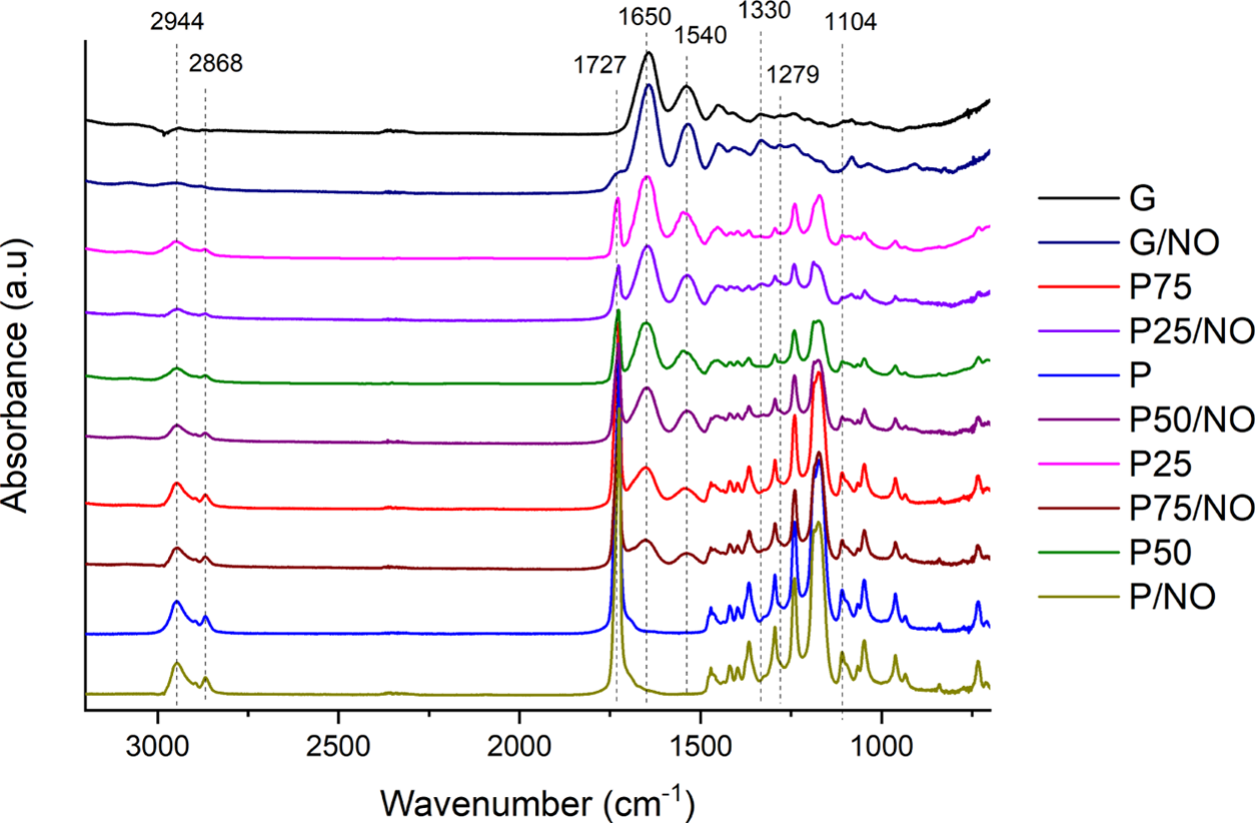
FTIR spectra of pure gelatin, PCL/gelatin, PCL, and NO release-functionalized membranes.

The FTIR spectra of the varying formulations of electrospun PCL/gelatin membranes are shown in Figure 4. The peaks at 2944 and 2868 cm^–1^ correspond to the stretching vibration of asymmetric and symmetric CH_2_ bonds from PCL, respectively.^39^ The peak at 1727 cm^–1^ is assigned to the carbonyl stretching of PCL.^40^ The peaks at ∼1650 cm^–1^ (amide I) and ∼1540 cm^–1^ (amide II) are assigned to the stretching vibration of the C=O bond and the coupling vibrations of N–H and C–N from gelatin, respectively.^28,41^ The diazeniumdiolate functionalization has been confirmed by the presence of N–N and N–O stretching at 909–1082 and 1279 cm^–1^ (Figure S2), respectively, and O–N–N–O deformation spectra peak at 1330–1405 cm^–1^ (Figure S2).^42^

### 3.5 Hydrolytic Degradation

**Figure 5:**
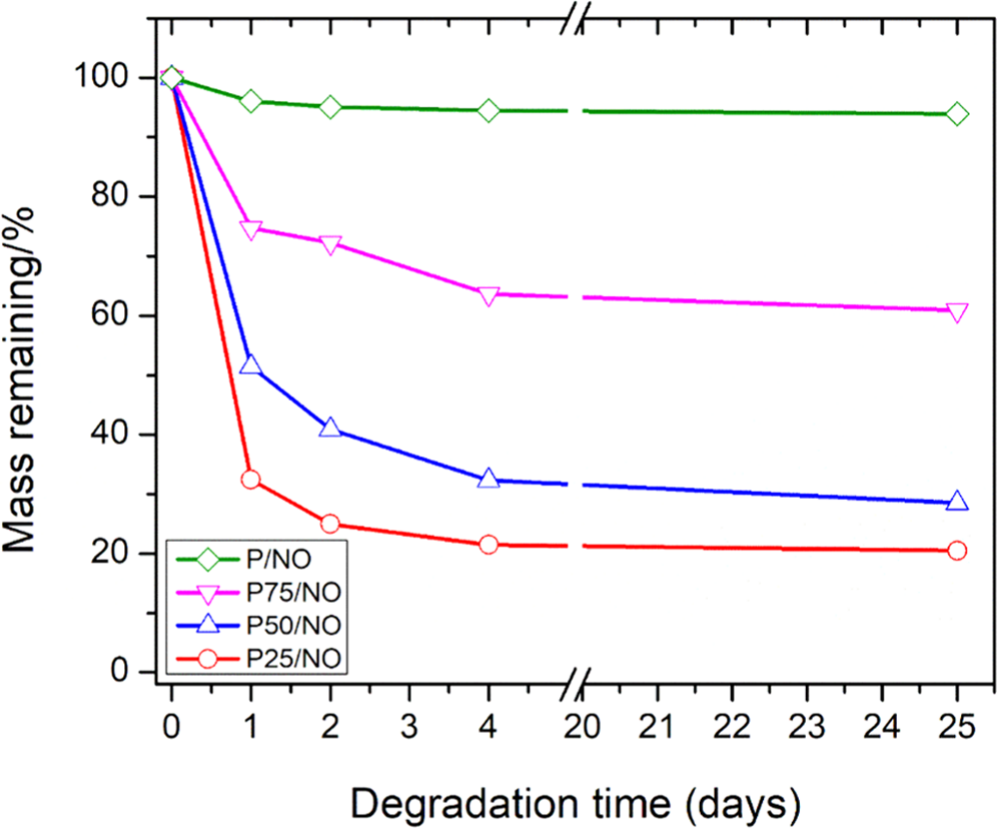
Degradation of the NO releasing membranes in PBS at 37 °C within 25 days.

Hydrolytic degradation of the PCL/G membranes is shown in Figure 5. The NO releasing membranes were soaked in PBS (pH = 7.4) for 25 days for hydrolytic degradation evaluation. P/NO remained at 94% of its primary weight until the 25th day. The P75/NO sample displayed a mass remaining of 75, 72, 64, and 61% at day 1, 2, 4, and 25, respectively. The P50/NO sample displayed a mass remaining of 51, 41, 32, and 29% at day 1, 2, 4, and 25, respectively. Finally, the P25/NO sample displayed a mass remaining of 32, 25, 21, and 20% at day 1, 2, 4, and 25, respectively. We have found that crosslinking slows down this hydrolytic degradation. This is in line with what has been observed by Correia et al., who have shown that weight loss for electrospun gelatin fibers occurs due to solvation and depolymerization of the main gelatin polymer chains. Crosslinking leads to a more disordered, insoluble, and rigid structure and the formation of hydrogen-bonded polymer networks, which enhances the mechanical properties.^43^ Indeed, the pure gelatin sample, G/NO, dissolved immediately and no time course degradation profile could be achieved.

### 3.6 Mechanical Properties

**Figure 6:**
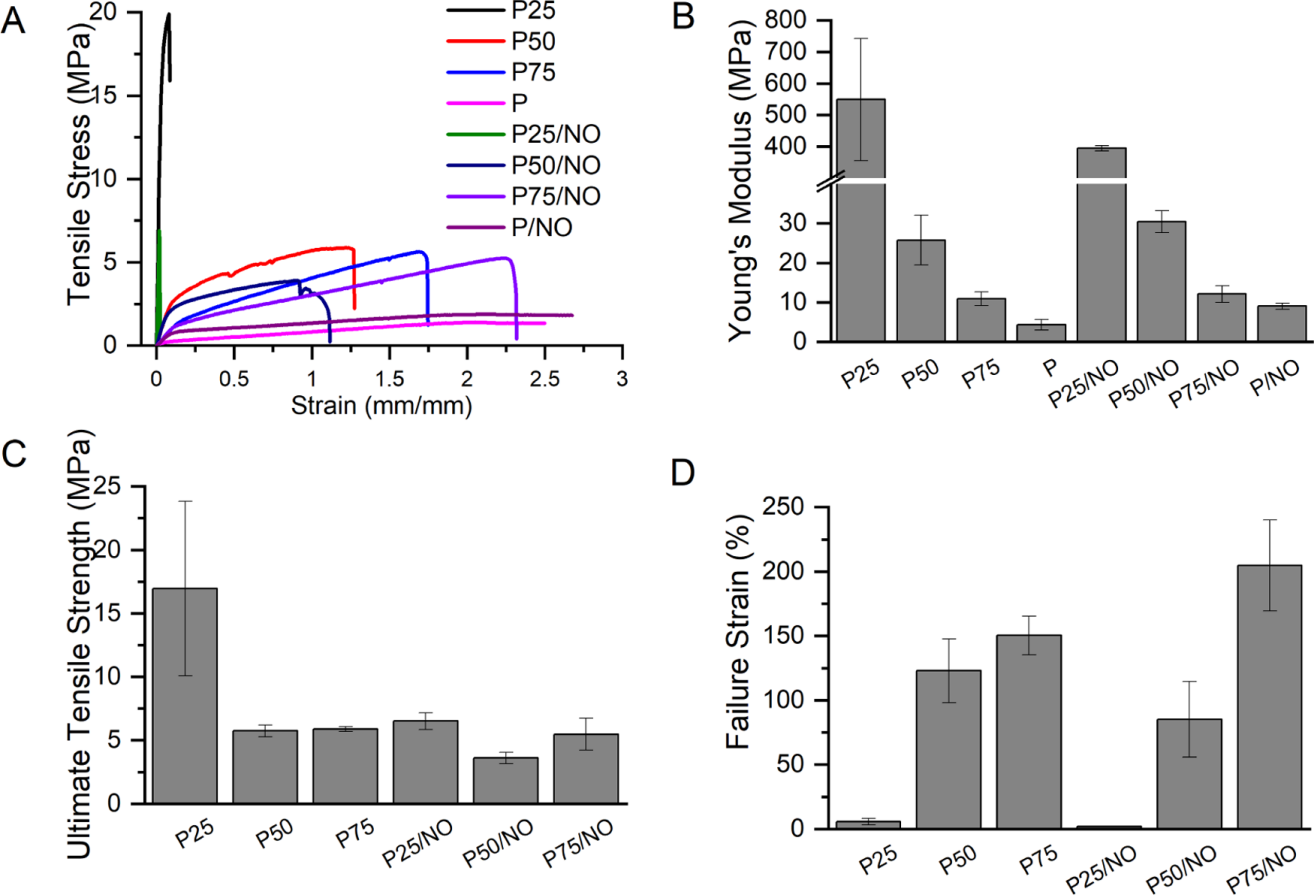
Mechanical properties of the NO releasing electrospun membranes: (A) tensile stress–strain curves, (B) Young’s modulus, (C) ultimate tensile strength, and (D) failure strain.

Wound dressings require the appropriate mechanical properties to function properly as brittle dressings can be easily damaged and would have to be replaced more frequently.^45^ The mechanical properties of the various samples were investigated, and the results are presented in Figure 6. Typical stress–strain curves for various PCL/G samples are given in Figure 6A. The concentration of gelatin in the membranes led to changes in mechanical properties, including higher Young’s modulus (Figure 6B), higher ultimate tensile strength (UTS) (Figure 6C), and lower break strain (Figure 6D). This was most likely due to the superior mechanical strength of PCL in comparison with G. The addition of gelatin into the polymer blend resulted in an increase in stiffness; this effect has been previously reported.^35,46^ G and G/NO samples are fragile in handling; therefore, these have not been included in the tensile testing. The P25 sample displayed the highest Young’s modulus of 549 MPa. With decreasing gelatin content, the Young’s Modulus decreases dramatically for P50 (25 MPa) and P75 (10 MPa) samples. The PCL/G samples displayed an elongation at maximum load in the range of 14–208% with a UTS range of 3.7–16.9 MPa. The UTS of the P25 sample was significantly different from all other samples (*p* < 0.0001); there were no other significant differences in UTS. Failure strain was dependent upon PCL content in the samples, with high PCL concentrations resulting in greater strains. These results are in agreement for previously tensile strengths 2.14 MPa;^47^ NO functionalization did not affect the mechanical properties of the membranes. The materials developed here have shown that changing of the mass ratios of PCL/G in membranes (for all formulations except pure gelatin, G and G/NO) gave materials with appropriate mechanical properties to shield the wound from a physical disruption and were contained in the ideal tensile strength range for skin cell culture and wound dressings (0.8–18 MPa).^47^

### 3.7 Chemiluminescence: Measurement and Mechanism of NO Release

*N*-Diazeniumdiolates formed on amine sites are widely investigated owing to their ease of synthesis, stability, and proton-initiated decomposition for NO release occurring at physiological conditions (Figure S3).^48,49^ It is widely accepted that efficient diazeniumdiolate formation occurs on secondary amines in situ under high pressures (4–5 atm) of NO in the absence of oxygen without the formation of nitrosamines. This has been previously observed by both the Keefer and Schoenfisch groups.^48−50^

**Figure 7:**
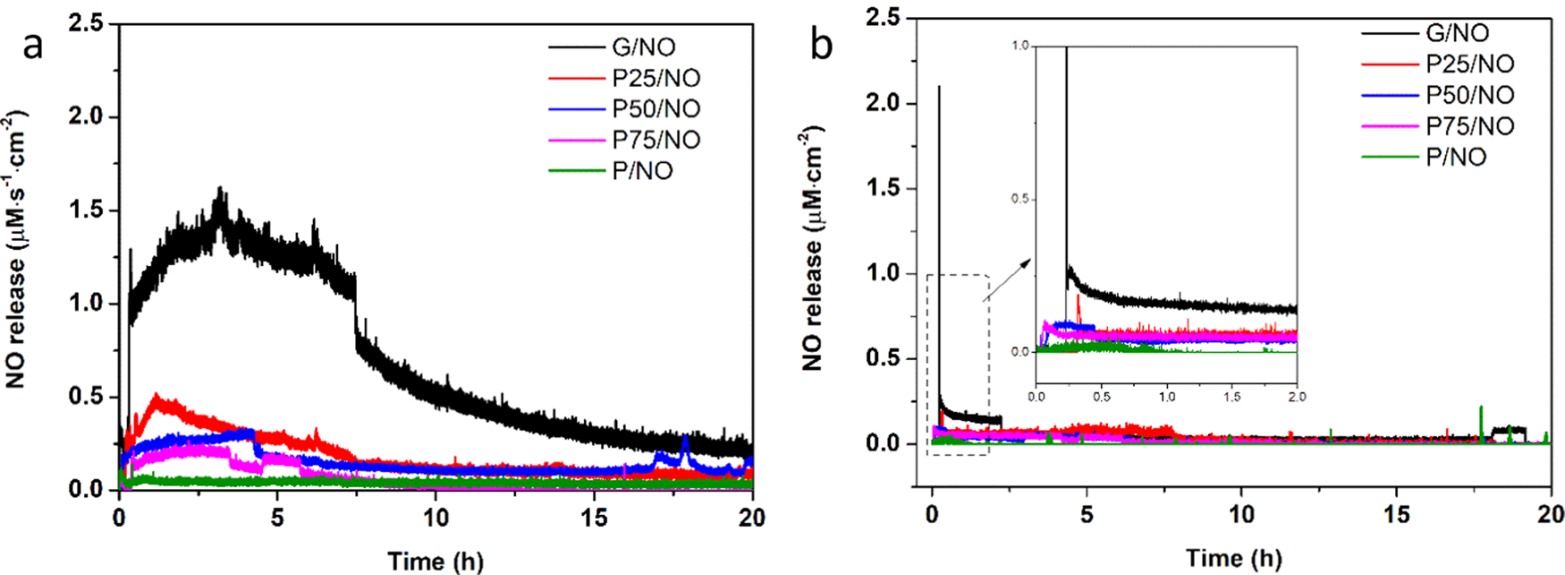
Chemiluminescence NO releasing profiles of the diazeniumdiolate-functionalized electrospun membranes at (a) pH 4 and (b) pH 7.4 at room temperature. The inset in (b) is rescaling of the first 2 h.

The NO payload from the various NO-releasing membranes was determined using a chemiluminescence nitric oxide analyzer at pH 4 (acetic acid buffer) and pH 7.4 (PBS). The NO-releasing profiles of electrospun membranes are shown in Figure 7. The kinetics of NO release are summarized in Table 3. These values include the total concentration of NO (*t*[NO]), maximum instantaneous concentration of NO released ([NO]_m_), the time needed to get to [NO]_m_ (*t*_m_), and the duration of NO release (*t*_d_), which were determined from each membrane.

**Table 3:** NO Releasing Properties of the Diazeniumdiolate-Functionalized Electrospun Membranes in pH 4 and 7.4

pH	membrane	*t*[NO] (mM)	[NO]_m_ (μM·s^–1^·cm^–2^)	*t*_m_ (min)	*t*_d_ (h)
4	G/NO	35.5	1.6	193	20+
	P25/NO	12.6	0.5	68	20+
	P50/NO	9.1	0.3	250	20+
	P75/NO	4.2	0.2	161	20+
	P/NO	0.3	0.1	4	
7.4	G/NO	3.0	2.1	14	10+
	P25/NO	2.1	0.2	20	10+
	P50/NO	1.1	0.1	16	8.4
	P75/NO	1.0	0.1	6	8.1
	P/NO	0.07	0.04	32	

G/NO: At pH 4, G/NO membranes displayed a maximum instantaneous NO concentration of 1.6 μM·s^–1^·cm^–2^, which was released at 193 min with a total NO release concentration of 35.5 mM within 20 h. At pH 7.4, the total NO released of G/NO was a smaller amount than that seen at pH 4. A total concentration of 3 mM and a maximum instantaneous NO concentration of 2.1 μM·s^–1^·cm^–2^ was observed.

P25/NO: At pH 4, P25/NO displayed a maximum instantaneous NO concentration of 0.5 μM·s^–1^·cm^–2^ at 68 min with a total NO release concentration of 12.6 mM within 20 h. At pH 7.4, the total NO release of P25/NO was a smaller amount than that seen at pH 4. A concentration of 2.1 mM and a maximum instantaneous NO concentration of 0.2 μM·s^–1^·cm^–2^ were observed.

P50/NO: At pH 4, P50/NO displayed a maximum instantaneous NO concentration of 0.3 μM·s^–1^·cm^–2^ at 250 min with a total NO release concentration of 9.1 mM within 20 h. At pH 7.4, the total NO release of P50/NO was a smaller amount than that seen at pH 4. A concentration of 1.1 mM and a maximum instantaneous NO concentration of 0.1 μM·s^–1^·cm^–2^ were observed.

P75/NO: At pH 4, P75/NO displayed a maximum instantaneous NO concentration of 0.2 μM·s^–1^·cm^–2^ at 161 min with a total NO release concentration of 4.2 mM within 20 h. At pH 7.4, the total NO release of P75/NO was a smaller amount than that seen at pH 4. A concentration of 1 mM and a maximum instantaneous NO concentration of 0.1 μM·s^–1^·cm^–2^ were observed.

P/NO: As there is no nitrogen in the backbone of PCL, it was expected that no diazeniumdiolate formation would take place and, hence, no NO release should be observed. A small amount of NO has been measured at both pH 4 and 7.4, which is presumed to be due to free NO gas molecules physisorbed on the surface of membranes.

From the results, it can be clearly seen that the NO payload and release kinetics corresponded to the gelatin content percentage of the membranes, the pH of the measuring buffers, and the crosslinking. The G/NO membranes released a higher total NO concentration than P25/NO at pH 4, and the same trend displayed at pH 7.4. Higher NO concentration was expected from the membranes at pH 4. This is because *N*-diazeniumdiolates that are formed on primary amine sites are known to break down to HNO and NO, with the ratio of the products depending on the pH of the solution.^51^ At pH 4, *N*-diazeniumdiolates are known to decompose solely to NO, and at pH 7.4, a mixture of HNO and NO is produced.

### 3.8 Antimicrobial Analysis

Planktonic bacteria are free-floating organisms and can colonize an open wound bed. Once these bacteria adhere to the surface, they can form biofilms. Biofilms are multicellular communities that secrete a protective extracellular polysaccharide matrix (EPS) around them, making them much more difficult to eradicate. Wounds that have been colonized by biofilms demonstrate delayed healing and can contribute to wound chronicity.^52,53^ James et al. have shown that in a histological analysis of clinical sample wounds, biofilms were found in 60% of chronic wound samples in comparison with 6% of acute wound samples.^53^ Therefore, an ideal wound dressing should prevent planktonic and biofilm-adhered bacteria. In this paper, the antimicrobial efficacy of the various electrospun NO-releasing membranes were investigated against a Gram-negative (*E. coli*) and a Gram-positive (*S. aureus*) bacteria, which are commonly found organisms in infected wounds.^54^

#### 3.8.1 Biofilm Inhibition

**Figure 8:**
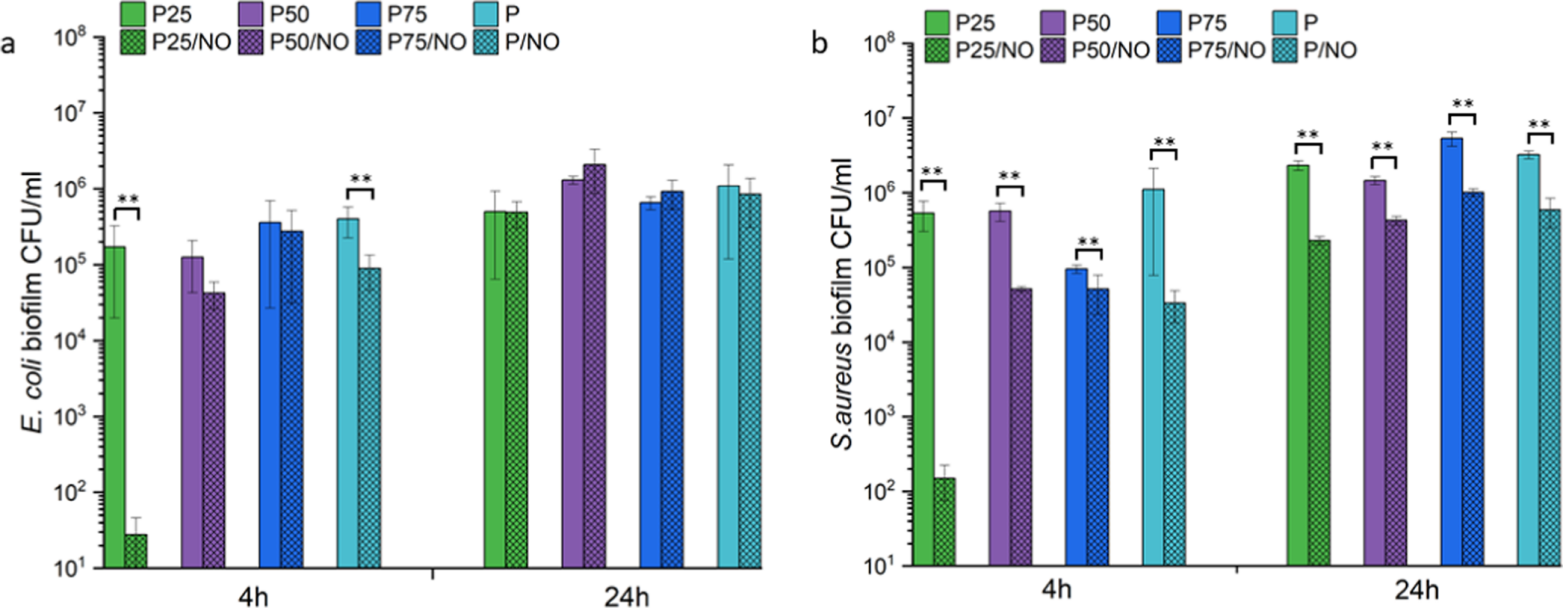
Viable bacterial colonies (CFU/mL) after 4 and 24 h of biofilm growth on electrospun membranes of (a) *E. coli* and (b) *S. aureus*. *p < 0.05 and **p < 0.01 from the corresponding ratio with no NO functionalization.

As biofilms are prolific in wounds and can prevent wound closure, eradicating them in the initial stages can speed up the wound-healing process.^55^ The prevention of biofilm formation on various NO-releasing membranes at 4 h was determined by biofilm CFU assay, with the results given in Figure 8. The G/NO membrane dissolved in the media after 4 h, therefore, biofilm formation on this sample was not possible. The P25/NO sample displayed the highest reduction of *E. coli* adhesion from 1.7 × 10^5^ to 2.7 × 10 CFU/mL, which corresponds to ∼4-log reduction (Figure 8a). The P50/NO and P75/NO membranes showed no significant difference in reduction of *E. coli* adhesion compared with the control samples. It can be clearly seen that higher gelatin concentration (>50%) largely improved the antimicrobial efficacy of P25/NO against *E. coli*, which is a consequence of increased NO payload. After 24 h, regrowth of all bacteria was observed.

The results of biofilm CFU assay against *S. aureus* on the membranes (Figure 8b) showed a similar trend with of that of *E. coli*. P25/NO membranes displayed a ∼3.3-log reduction (*S. aureus* biofilm decreased from 4 × 10^5^ to 1.5 × 10^2^ CFU/mL), which corresponds to the highest reduction for all of the membranes at the 4 h incubation time point. The P50/NO, P75/NO, and P/NO membranes showed 1–1.5 log reduction in *S. aureus* adhesion at 4 h. At the 24 h timepoint, whilst some regrowth of *S. aureus* has been found, a ∼1-log reduction of biofilm formation is still observed on all of the NO-releasing membranes investigated.

For a material to be bactericidal, there needs to be at least 3 orders of magnitude reduction^56^ in bacterial counts, and the P25/NO formulation displays an antibiofilm bactericidal activity against both adhered *E. coli* and *S. aureus*, common bacteria found in wounds.^54^ The P50/NO, P75/NO, and P/NO membranes with lower NO concentrations displayed a moderate activity against biofilm-adhered *S. aureus* bacteria with a reduction of between 1 and 2 log. NO’s efficacy as an antibiofilm agent can be attributed to the fact that it is not reliant on any metabolic process, but as it is a small, uncharged molecule that is able to easily permeate through a biofilm matrix killing adhered bacteria.

#### 3.8.2 Antimicrobial Activity on Planktonic Bacteria

**Figure 9:**
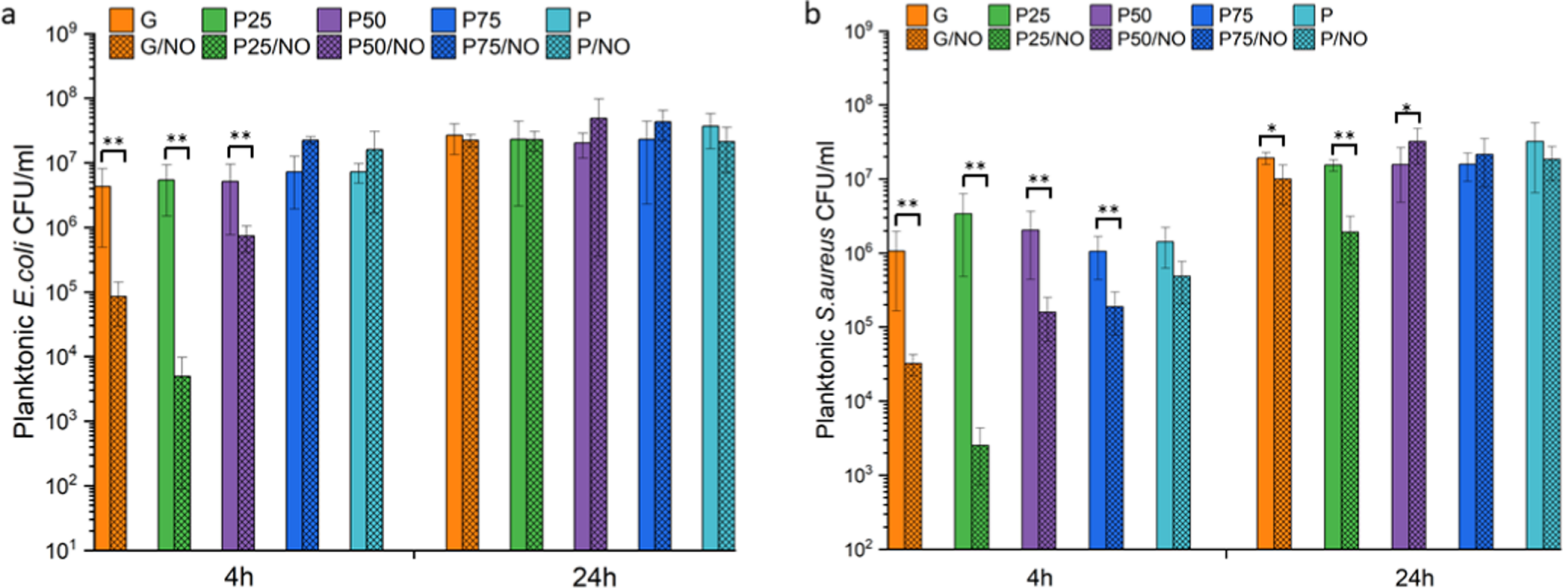
Planktonic (a) *E. coli* and (b) *S. aureus* growth in solution with the presence of the electrospun membranes after 4 and 24 h of incubation. *p < 0.05 and **p < 0.01 from the corresponding ratio with no NO functionalization.

The growth of planktonic bacteria at 4 h on the electrospun membranes is shown in Figure 9. The G/NO membrane displayed a reduction of planktonic *E. coli* growth from 4.3 × 10^6^ to 8.5 × 10^4^ CFU/mL, which corresponds to a ∼1.5-log reduction (Figure 9a). P25/NO displayed the highest reduction of planktonic *E. coli* proliferation corresponding to ∼3-logs. The P50/NO, P75/NO, and P/NO membranes showed less than 1-log reduction in planktonic *E. coli* growth. G/NO and P25/NO had the highest antimicrobial efficiency, and this corresponds to an increase in the payload of NO due to the increased amine/amide functional groups in the blends.

The results against *S. aureus* (Figure 9b) showed a similar trend as against *E. coli* on various membranes at 4 h incubation. The P25/NO membrane displayed the highest reduction of planktonic *S. aureus* growth from 3.4 × 10^6^ to 2.5 × 10^3^ CFU/mL, which corresponds to a ∼3-log reduction. The G/NO membrane displayed a 1.5-log reduction of planktonic *S. aureus* growth. P50/NO, P75/NO, and P/NO showed about 1-log reduction from 1.5 × 10^7^ to 1.9 × 10^6^ CFU/mL in planktonic *S. aureus* growth. At 24 h, bacteria have regrown; however, G/NO showed a reduction of ∼50% (1.9 × 10^7^ to 1 × 10^7^ CFU/mL) and P25/NO gave a ∼90% reduction (1.5 × 10^7^ to 1.9 × 10^6^ CFU/mL). Planktonic *S. aureus* has previously been observed to be more susceptible to NO exposure than *E. coli*.^57^ This is possibly due to the fact that Gram-negative bacteria such as *E. coli* have flavohemoglobins that can neutralize NO and therefore can attenuate bacterial kill.^58^

Similar to what was observed for the biofilm-adhered bacteria, the P25/NO membrane displayed bactericidal activity (3-log reduction) after 4 h against *E. coli* and *S. aureus* planktonic bacteria. P50/NO, P75/NO, and P/NO displayed moderate activity corresponding to a 1–3 log reduction against *S. aureus* at 4 h.

### 3.9 Cell Viability Assay

**Figure 10:**
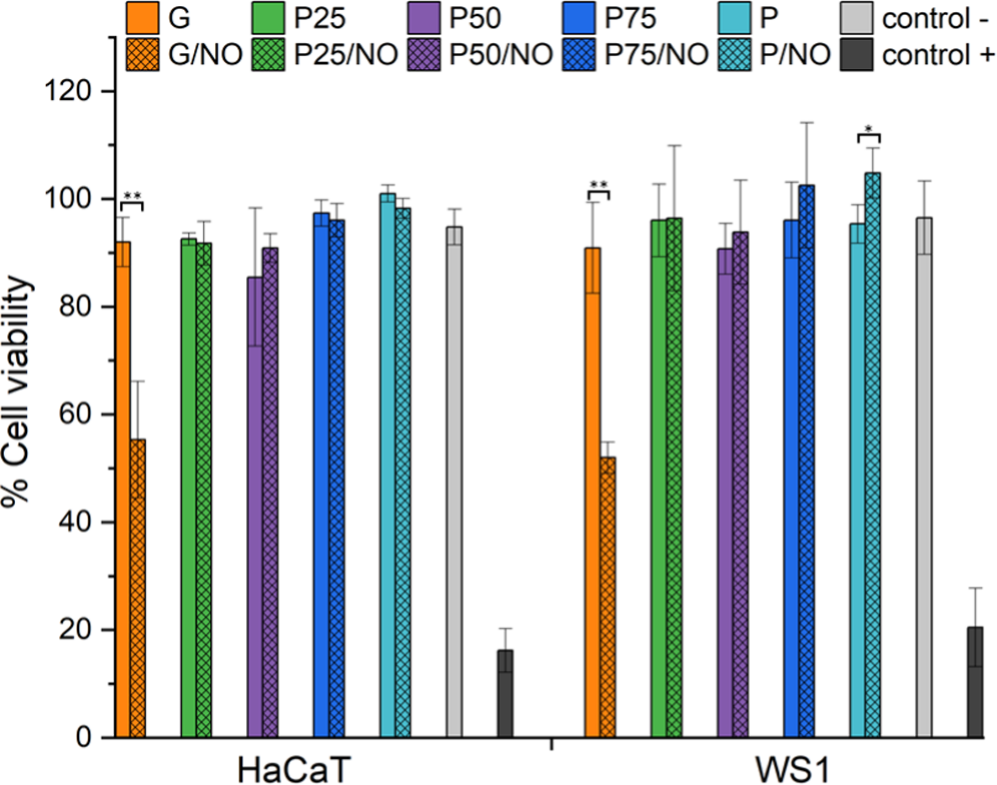
Cytotoxicity of electrospun membranes on HaCaT and WS1 cells after 24 h. *p < 0.05 and **p < 0.01 from the corresponding ratio without NO functionalization.

Wound dressing materials need to be biocompatible and noncytotoxic. The assessment of *in vitro* cytotoxicity represents the initial step in the biocompatibility evaluation.^59^ In this study, a resazurin assay was used to evaluate the biocompatibility of the various membranes before and after diazeniumdiolate functionalization via a leaching assay according to ISO10993-5 (Figure 10). The membrane leachables were extracted in cell culture media for 24 h. HaCaT keratinocytes and WS1 fibroblasts were cultured in conditioned media, and a resazurin assay was performed after 24 h in culture. The NO-releasing profile of the membranes in cell media (shown in Figure S4) was slightly different than when carried out in PBS. The maximum instantaneous NO concentration [NO]_m_ of G/NO in cell culture media was 0.8 μM·s^–1^·cm^–2^, compared to 0.2 μM·s^–1^·cm^–2^ in PBS (pH 7.4). The G/NO releases a total NO (*t*[NO]) of 2.0 mM for over 20 h in cell culture media compared with a total NO amount of 3.0 mM over 10 h in PBS. The maximum instantaneous NO concentration ([NO]_m_) of P25/NO in cell culture media was 0.3 μM·s^–1^·cm^–2^, compared to 0.2 μM·s^–1^·cm^–2^ in PBS (pH 7.4). P25/NO releases a total NO (*t*[NO]) of 1.8 mM for over 24 h in cell culture media compared to a total NO amount of 2.1 mM over 10 h in PBS. While NO has been detected at 24 h in cell culture media, it is present at a very low concentration. Furthermore, no cytotoxic effects were observed for any of the sample types except for G/NO. The cytotoxicity of this sample is due to the cumulative release of a large concentration of NO (2 mM, Table S1). Previous work by Maloney et al. has shown that cytotoxic NO doses occur between 1 and 3.3 mM.^60^ Although the P25/NO (1.8 mM) and P50/NO (1.0 mM) samples release NO at a concentration within this cytotoxic range as the release profile is slower, there is no rapid generation of cytotoxic concentrations of NO as observed for the G/NO sample.

### 3.10 Wound Gap Closure Assay

**Figure 11:**
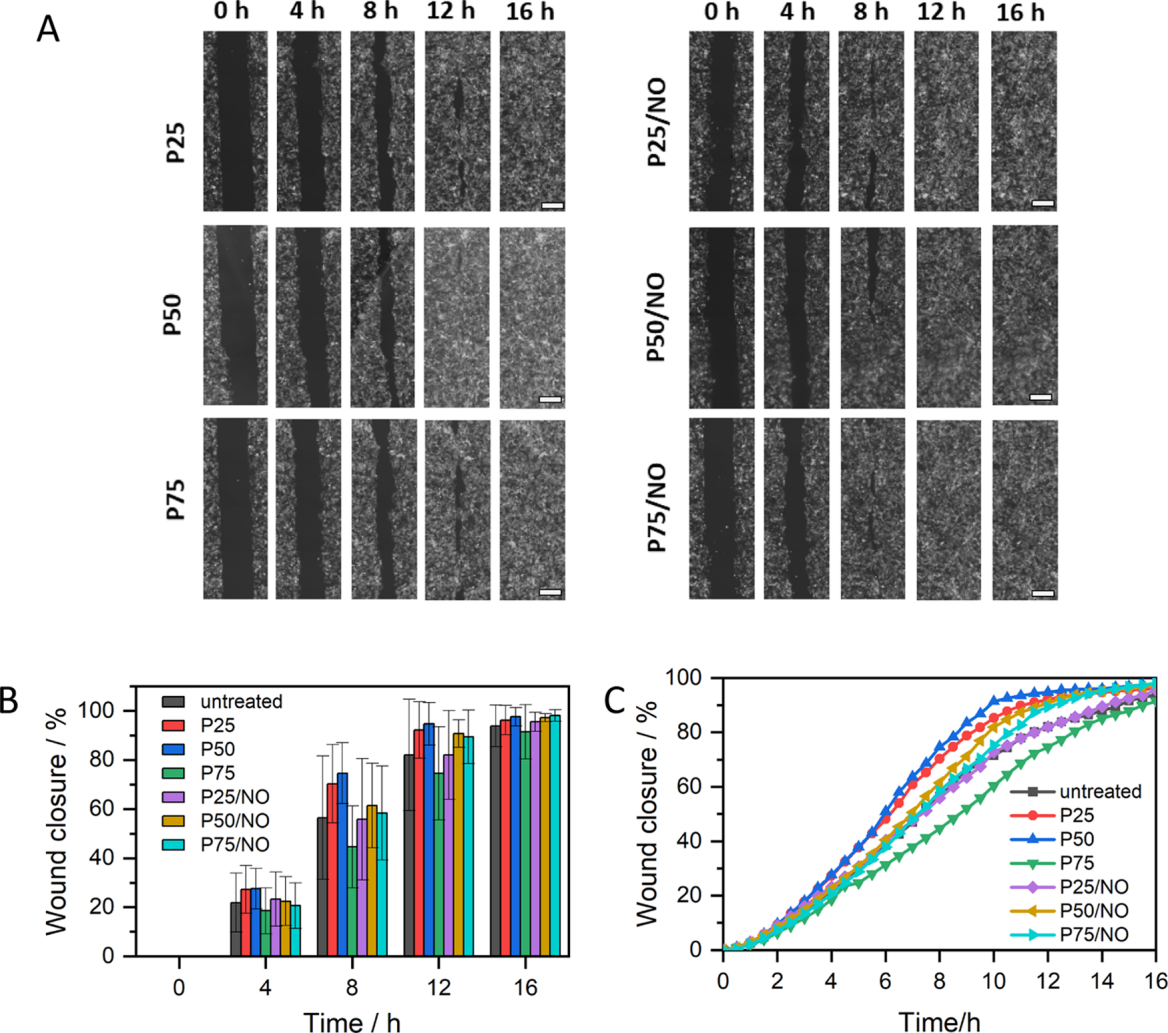
(A) Fluorescent images of HaCaT cells cultured on P25, P50, P75, P25/NO, P50/NO, and P75/NO at different time points, scar bar represents 300 μm; (B) percentage of wound closure at specific time points of HaCaT cells over a 16 h time period; and (C) a summary wound gap closure of HaCaT cells over 16 h time period.

Wound healing is a multistep process that involves the attachment, proliferation, and migration of cells to regenerate new tissue. A simple and cost-effective method to study this process is via an *in vitro* scratch assay. In this experiment, we carried out an indirect analysis to investigate whether any of the leachates from NO-releasing membranes had a negative effect on the wound repair pathway. The NO-releasing membranes were exposed to cell culture media for 24 h, and this media with the leachates were then used to challenge HaCaT cells. Cells were cultured in the leachate-containing media and grown for a 16 h time period, as shown in Figure 11. The corresponding time-lapse videos for the wound closure experiment are given in the Supporting Information (Video 1: P25, Video 2: P25/NO, Video 3: P50, Video 4: P50/NO, Video 5: P75, Video 6: P75/NO, Video 7: Tissue Culture Polystyrene Control). There were no significant differences in percentage gap closure between samples or compared to an untreated control at 4, 8, 12, or 16 h after gap creation. All samples had a gap closure of >90% by 16 h. These results demonstrate that none of the NO released byproducts have had an effect on wound closure rates. An accelerated rate of wound closure was not observed for NO-releasing samples, but this is to be expected as all of the NO would have dissipated after 24 h. The benefit of NO as a wound-healing agent is the subject of ongoing work, where it will be studied in a direct contact assay as well as a bacterial-cell co-culture model, where NO’s antibiofilm and bactericidal activity can target the infection and perhaps have a positive effect on wound healing.

## 4 Conclusions

We report on the fabrication and the antimicrobial efficacy of five formulations of electrospun PCL/G membranes. The membranes were successfully electrospun into uniform bead-free nanofibers, and genipin was used to crosslink the membranes to increase its stability. The resultant membranes demonstrated appropriate mechanical properties, biodegradation, and biocompatibility for use as a wound dressing. The antimicrobial efficacy of the membranes was directly correlated with the mass ratio of gelatin in electrospun membranes. The higher the gelatin in the membrane, the greater the NO loading, which lead to higher antimicrobial efficiency against *E. coli* and *S. aureus*. The *in vitro* wound-healing assay demonstrated that NO-releasing membranes had no cytotoxic effect on the wound healing progression, but they do have the ability to potentially eradicate the initial stages of an infection on a wound. In developing an antimicrobial dressing, ideally the material should have minimal cytotoxic effects to the host with an easy dosing regimen and bactericidal rather than bacteriostatic action. Overall, the materials developed here are noncytotoxic and have demonstrated bactericidal and antibiofilm action against common wound pathogens. Therefore, these membranes have excellent potential to be developed into antimicrobial wound dressings.

## Supporting Information Available

Diameter distribution for each crosslinked electrospun (Figure S1); FTIR spectra of c_P25 and c_P25/NO membranes (Figure S2); mechanism of diazeiumdiolate formation (Figure S3); chemiluminescence of NO release profiles (Figure S4); and NO release properties for diazeniumdiolate-functionalized membranes (Table S1) (PDF)
Video 1: P25 (AVI)
Video 2: P25/NO (AVI)
Video 3: P50 (AVI)
Video 4: P50/NO (AVI)
Video 5: P75 (AVI)
Video 6: P75/NO (AVI)
Video 7: Tissue Culture Polystyrene Control (AVI)

## Supplementary Material

mg1c00056_si_001.pdf

mg1c00056_si_002.avi

mg1c00056_si_003.avi

mg1c00056_si_004.avi

mg1c00056_si_005.avi

mg1c00056_si_006.avi

mg1c00056_si_007.avi

mg1c00056_si_008.avi
