# Supplementary material for: Antimicrobial Nitric Oxide-Releasing Electrospun Dressings for Wound Healing Applications
Source: ACS Mater Au. 2022 Jan 25;2(2):190–203. doi: 10.1021/acsmaterialsau.1c00056 (PMC9888637; doi:10.1021/acsmaterialsau.1c00056)
Supplement: Supplementary file 1 — mg1c00056_si_001.pdf [file mg1c00056_si_001.pdf]

Antimicrobial nitric oxide releasing electrospun dressings for wound healing applications

Man Li[1], Jenny Aveyard[1], Kyle Doherty[2], Robert C. Deller[1], Rachel L. Williams[2], Keli N. Kolegraff[3], Stephen B. Kaye[2] and Raechelle A. D'Sa[1,*]

[1] School of Engineering, University of Liverpool, Liverpool, L69 3GH, United Kingdom

[2] Department of Eye and Vision Science, Institute of Life Course and Medical Science, University of Liverpool, L7 8TX, United Kingdom

[3]Department of Plastic and Reconstructive Surgery, The Johns Hopkins University School of Medicine, 601 North Caroline Street, Baltimore, Maryland 21287, United States

[*]Corresponding Author: r.dsa@liverpool.ac.uk

SUPPORTING INFORMATION

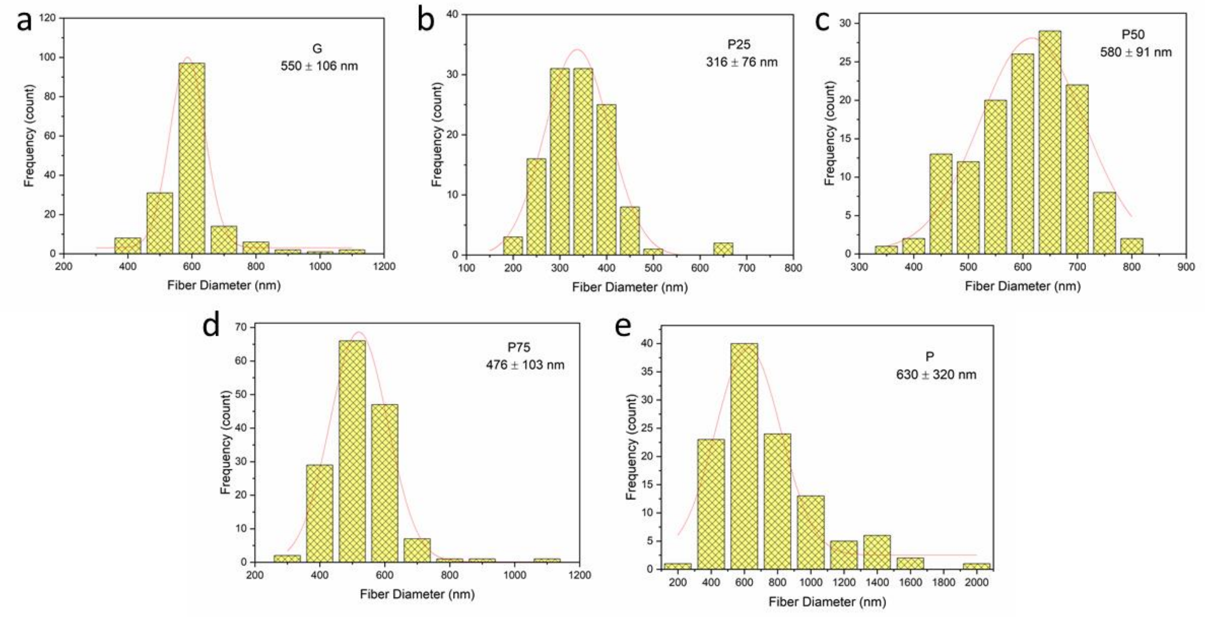

Figure S1. Diameter distribution for each crosslinked electrospun PCL/GT membranes a) c_G, b) c_P25, c) c_P50, d) c_P75, e) c_P. At least 115 randomly selected fibers in multiple micrographs were measured in ImageJ to obtain the diameter distribution.

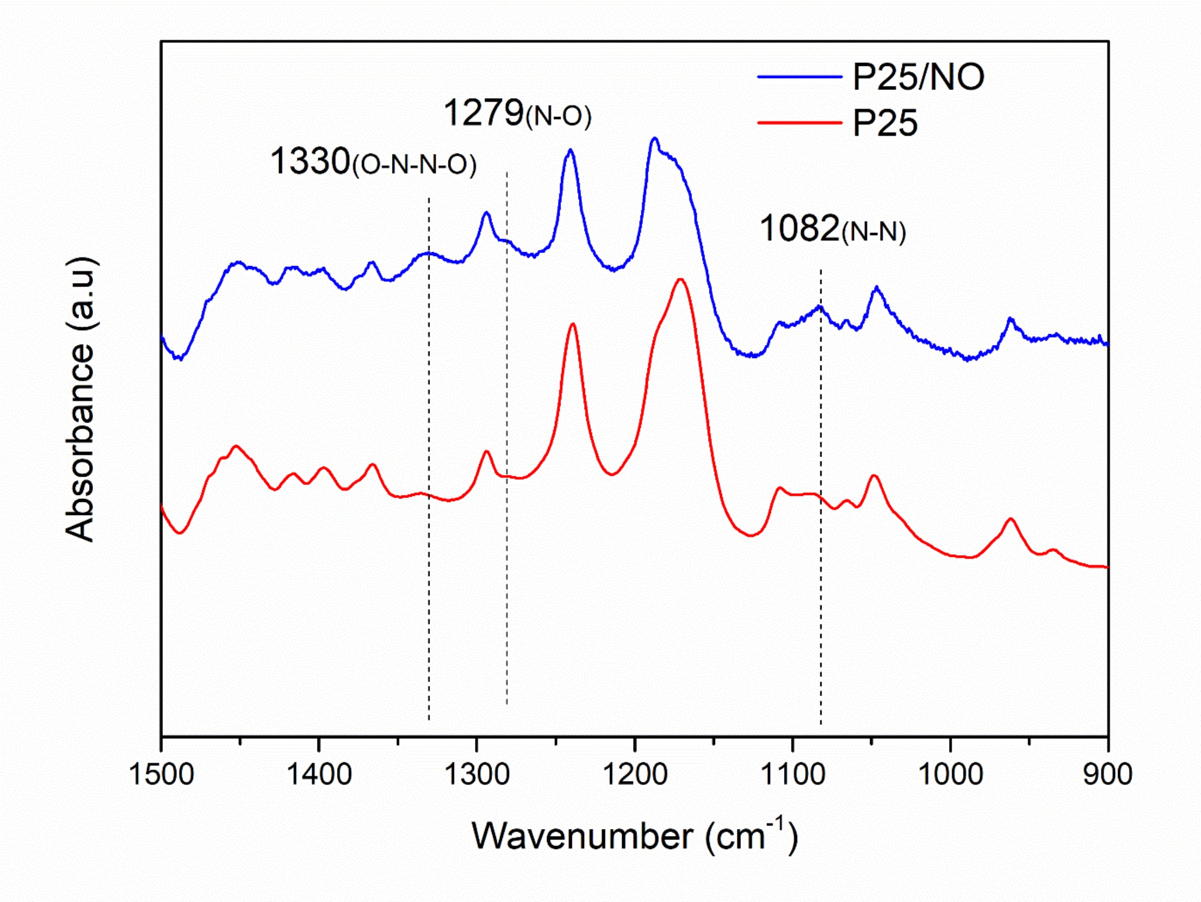

Figure S2. FTIR spectra of c_P25 and c_P25/NO membranes. Diazeniumdiolate functionalization was confirmed by the appearance of characteristic peaks at 909-1082, ~1279 and 1330-1405 cm^{-1}.

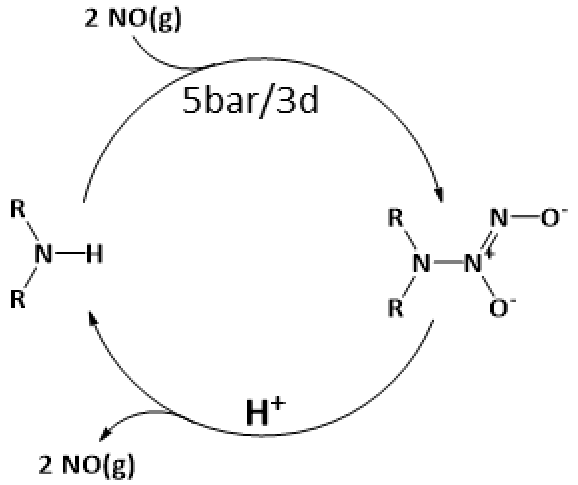

Figure S3. Mechanism of Diazeiumdiolate formation and decomposition.

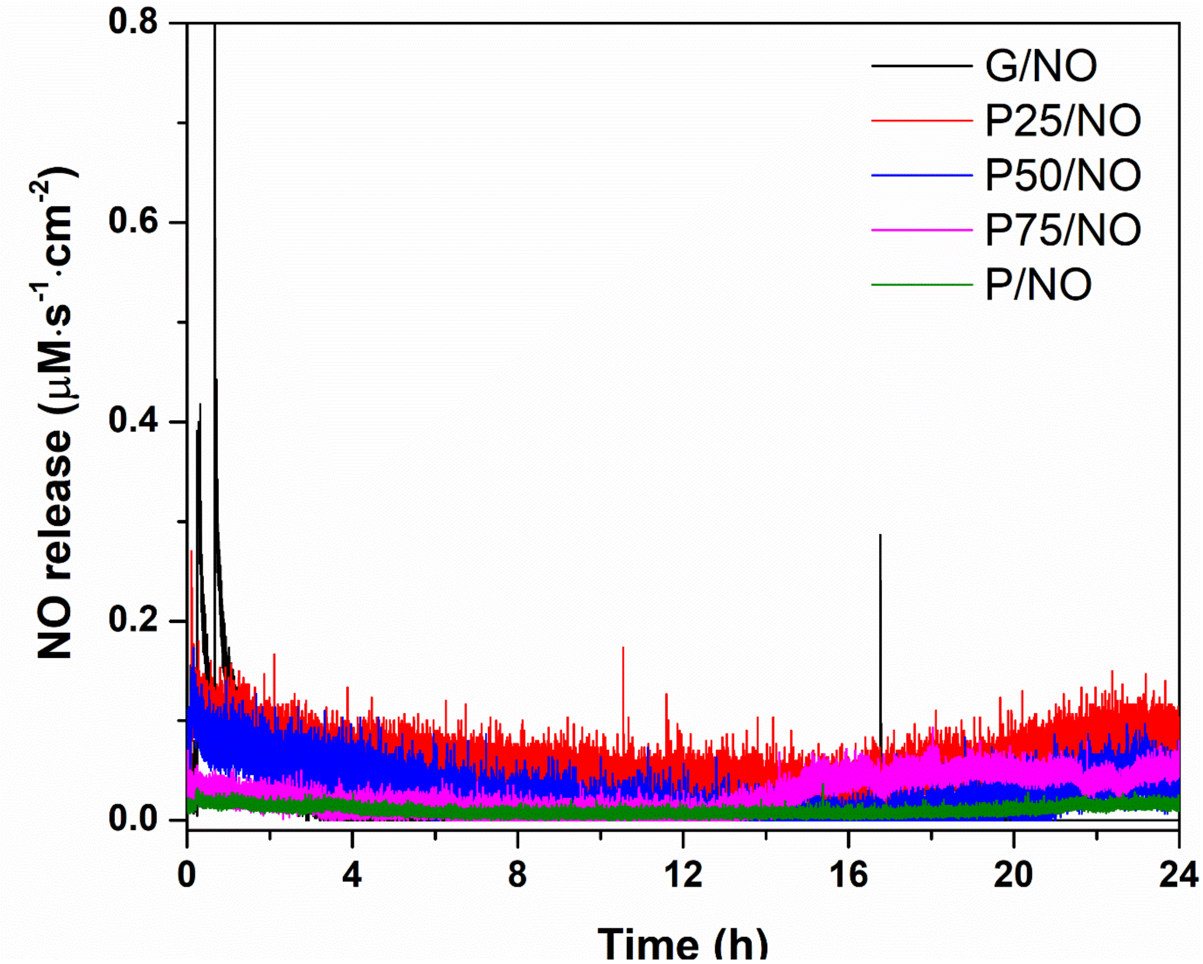

Figure S4: Chemiluminescence NO release profiles of diazeniumdiolate-functionalised membranes in cell culture medium at room temperature over time period of 24 h.

Table S1. NO release properties for diazeniumdiolate-functionalised membranes in cell culture medium.

membrane	t[NO] (mM)	$[NO]_m$ ($\mu M \cdot s^{-1} \cdot cm^{-2}$)	t_m (min)	t_d (h)
G/NO	2.0	0.8	38	20+
P25/NO	1.8	0.3	8	24+
P50/NO	1.0	0.1	11	24+
P75/NO	0.5	0.1	-	24+
P/NO	0.04	-	-	-
